# Valorization of Artichoke Bracts in Pasta Enrichment: Impact on Nutritional, Technological, Antioxidant, and Sensorial Properties

**DOI:** 10.3390/antiox14040475

**Published:** 2025-04-16

**Authors:** Anna Rita Bavaro, Palmira De Bellis, Vito Linsalata, Serena Rucci, Stefano Predieri, Marta Cianciabella, Rachele Tamburino, Angela Cardinali

**Affiliations:** 1 Institute of Sciences of Food Production (ISPA), National Research Council (CNR), Via G. Amendola 122/O, 70126 Bari, Italy; annaritabavaro@cnr.it (A.R.B.); vito.linsalata@cnr.it (V.L.); serena.rucci@ispa.cnr.it (S.R.); angela.cardinali@cnr.it (A.C.); 2 Institute of BioEconomy (IBE), National Research Council (CNR), Via P. Gobetti 101, 40129 Bologna, Italy; stefano.predieri@ibe.cnr.it (S.P.); marta.cianciabella@ibe.cnr.it (M.C.); rachele.tamburino@ibe.cnr.it (R.T.)

**Keywords:** *Cynara cardunculus* var. *scolymus*, food by-products, outer bracts, enriched pasta, free and bound polyphenols, functional foods, high-fiber product, bioaccessibility, circular economy

## Abstract

The incorporation of artichoke bracts, a by-product of artichoke processing, into pasta formulations represents an innovative approach to enhancing the nutritional and functional properties of this staple food while promoting environmental sustainability. This study aimed to evaluate the impact of artichoke powder (AP) enrichment (10% *w*/*w* replacement of semolina) on the technological, nutritional, antioxidant, and sensory properties of pasta. The enriched pasta (P-AP) was compared to control pasta (P-CTR) through comprehensive physicochemical analyses, including cooking performance, polyphenol characterization, and in vitro digestion. Polyphenol analysis revealed that chlorogenic acid, dicaffeoylquinic acids, and flavonoids accounted for 87% of total identified phenolic compounds in P-AP. Despite a 42% reduction in free polyphenols due to cooking, in vitro digestion revealed a 47% increase in total identified polyphenols, attributed to the release of bound polyphenols. Antioxidant assays (DPPH, ABTS, and FRAP) confirmed a significantly higher antioxidant capacity in P-AP compared to P-CTR. Additionally, P-AP exhibited a lower predicted glycemic index (pGI = 56.67) than the control (pGI = 58.41), a beneficial feature for blood glucose regulation. Sensory analysis highlighted distinct differences between samples, with P-AP showing stronger vegetal, artichoke, and legume-like notes, as well as higher intensity in bitterness and astringency. While panelists rated P-CTR higher in overall liking, enriched pasta maintained acceptable sensory characteristics. These findings support the valorization of artichoke by-products in pasta production, demonstrating their potential to enhance nutritional quality and functional properties while contributing to a circular economy.

## 1. Introduction

In recent years, interest in functional foods has increased significantly, leading to rapid growth in their global market [1]. These products were first defined in 1984 by the Japanese government as “Foods for Specified Health Use” (FOSHU), described as “foods containing an ingredient with health functions and officially approved to claim their physiological effects on the human body”. Functional foods offer significant benefits for both physical and mental well-being, which are key factors driving consumer acceptance. Consequently, the growing demand for these products has encouraged research on the incorporation of bioactive compounds into everyday foods [2]. Among these compounds, polyphenols and dietary fiber have gained particular attention for their well-documented health benefits, including antioxidant, anti-inflammatory, and gut health-promoting properties [3,4].

Artichoke (*Cynara cardunculus* var. *scolymus*), a vegetable typical of the Mediterranean Diet, is a rich source of polyphenols and fiber, making it highly valuable nutritionally [5]. It is cultivated mainly in the Mediterranean region, the Canary Islands, Egypt, Asia, and South America, with Italy, Spain, and France being the leading producers. The edible portion of the artichoke, its immature flower, represents about 30% of its fresh weight. Consequently, industrial processing generates 60–85% of bio-waste and by-products, including leaves, outer bracts, and stems, raising concerns about food waste and environmental impact [6]. Artichoke heads, leaves, and stems are rich in bioactive compounds, including caffeic acid derivatives, flavonoids (e.g., luteolin and apigenin), and dietary fiber such as inulin and pectins. These compounds confer on artichoke strong antioxidant activity, which is associated with protective effects against cardiovascular, hepatic, and neurological disorders [7]. As a result, artichoke by-products can be considered valuable ingredients for functional foods, supplements, and animal feed, while their reuse exemplifies a promising circular economy approach aimed at reducing the risk of metabolic and age-related disorders [8]. Several studies have demonstrated the successful incorporation of artichoke by-products into both non-food and food matrices, such as bread and pasta, to enhance their health-promoting properties [6,9,10].

The incorporation of agricultural and food industry residues into staple foods such as pasta offers a dual benefit as follows: reducing food waste and improving the nutritional profile of widely consumed products. Carpentieri et al. [11] conducted an extensive analysis of the use of bioactive compounds derived from agri-food by-products, such as dietary fibers, proteins, antioxidants, and omega-3 fatty acids, for pasta functionalization. Their review explored how these compounds influence the pasta-making process, as well as its technological, structural, sensory, and nutritional properties. Moreover, they discussed technological strategies, including sustainable emerging technologies, to mitigate the negative impacts of unconventional ingredients and enhance the health-promoting properties of pasta.

Among agri-food by-products, Colombo et al. [8] reported technological applications involving artichoke by-products, particularly outer bracts and stems. Most studies have focused on bakery products, such as bread and breadsticks, where artichoke powder is used as a partial substitute for conventional flour in varying proportions [10,12,13,14,15]. These studies assessed the effects of enrichment from technological, nutritional, and sensory perspectives. However, few studies have addressed pasta enrichment, and these typically involve the use of polyphenol extracts as a water substitute, enriching the pasta only with specific bioactive compounds, such as polyphenols, to enhance some properties, like antioxidant activity [16,17]. The literature contains very few studies utilizing whole artichoke by-products. Among these, the study by la Gatta et al. [18] stands out, as they investigated the use of a powder derived from artichoke outer bracts in fresh pasta formulation. This study evaluated the effects from a technological perspective, including protein interactions, as well as nutritional aspects such as glycemic index modulation and sensory characteristics influenced by the presence of volatile compounds typical of the vegetable matrix. Nonetheless, significant knowledge gaps remain regarding the effects of cooking and digestion on bioactive compounds, particularly polyphenols and fiber, their interactions with metabolism, and their potential health benefits for humans.

This study aims to investigate the feasibility of using artichoke by-products, such as outer bracts, to produce pasta enriched with polyphenols and fiber. By incorporating these bioactive compounds into pasta, a widely consumed product, its functional properties can be enhanced, potentially offering health benefits to consumers while promoting environmental sustainability. The research examined the impact of artichoke bract incorporation on pasta’s technological properties, nutritional quality, antioxidant activity, and sensory attributes.

## 2. Materials and Methods

### 2.1. Raw Materials

Fresh artichokes *(Cynara scolymus* (L.) var. *Romanesco*) were purchased from a local market (Bari, Italy) and used to obtain artichoke by-products. Specifically, in this study, the external bracts were collected and processed to produce artichoke powder (AP), as previously described by Bavaro et al. [10]. Commercial re-milled durum wheat semolina (Casillo, Corato, Italy) was also purchased from a local market. According to the label, semolina contained 2% total fat, 70% of total available carbohydrates, 2.8% total dietary fiber, and 12% crude proteins.

### 2.2. Pasta Making

The pasta was prepared by mixing semolina and water using a mixer (Pasta Mixer, Marcato, Campodarsego, Italy) equipped with accessories for Fettuccine production. This process yielded a dough with a moisture content of 44% (U%). The experimental dough was prepared by replacing 10% of the semolina with AP. A control sample (P-CTR) was produced using 100% re-milled durum wheat semolina. Fresh Fettuccine, approximately 20 cm long and 1.2 mm thick, was produced. The samples were then dried at 40 °C for 16 h using the Biosec dehydrator (Tauro Essiccatori, Camisano Vicentino, Italy) to achieve a moisture content <12.5%, in compliance with Italian legal requirements for dry pasta [19]. The samples were subsequently stored at room temperature under vacuum. Two different batches were produced. The experimental pasta (P-AP) and the control pasta (P-CTR) were subjected to technological, chemical, biological, and sensory analyses.

### 2.3. Physicochemical Characterization

The water-holding capacity of the re-milled durum wheat semolina and AP was determined as previously described [10] and expressed as the quantity of water held by 1 g of semolina or AP. The pH of pasta doughs was measured with a portable pH meter (type 110, Eutech Instruments, Singapore City, Singapore) supplied with a Double Pore D electrode (Hamilton, Bonaduz, Switzerland). Total titratable acidity (TTA) values of pasta doughs were determined according to American Association for Clinical Chemistry method (AACC) 02-31.01 [20]. Water activity (a_w_) was measured with AcquaLab (Decagon Devices, Inc., Pullman, WA, USA).

### 2.4. Technological Characterization of Pasta

The technological properties of both pasta samples were assessed following the methods described by Schettino et al. [21].

#### 2.4.1. Optimal Cooking Time

The optimal cooking time (OCT) of the pasta samples was determined according to the American Association of Cereal Chemists (AACC) approved method 66-50 [22]. Pasta samples were boiled in a 1:10 (*w*/*v*) dry pasta/water ratio without adding salt. Every 30 s, some Fettuccine strands were removed, cut, and examined for the disappearance of the white core.

#### 2.4.2. Water Absorption

Water absorption at the OCT was measured by weighing the pasta before and after cooking. It was calculated as ((W_1_ − W_0_)/W_0_) × 100, where W_1_ is the weight of the cooked pasta and W_0_ is the weight of the uncooked sample.

#### 2.4.3. Cooking Loss

Cooking loss was determined by measuring the amount of solid matter lost into the cooking water. Portions of 30 g of pasta were cooked in 300 mL of boiling tap water without salt. The pasta samples were cooked for the OCT, and the water volume was brought back to its initial level after cooking. Dry matter in the cooking water was determined from 25 mL of freeze-dried water residue. The residue was weighed and expressed as a percentage of dry material, reported as grams of matter lost per 100 g of pasta.

#### 2.4.4. Hydration Test

Five grams of pasta were placed in beakers containing 100 mL of tap water and incubated in a thermostatic bath at 25 °C. After 5, 10, 15, 30, 60, 90, and 180 min of incubation, each aliquot was removed from the water, drained for approximately 1 min, blotted with tissue paper, and weighed. The results were expressed as ((W_1_ − W_0_)/W_0_) × 100, where W_1_ represents the weight of the hydrated sample and W_0_ represents the weight of the dry sample.

#### 2.4.5. Instrumental Color

Color values were measured on ground pasta samples by a colorimeter (CR-400, Konica Minolta, Osaka, Japan) with a D65 illuminant, using attachment for granular materials CR-A50. The values were obtained using CIE color system coordinates L (lightness), a* (red-green), and b* (yellow-blue) on five measurements for each sample. Color difference, ∆E*ab, was calculated using the following formula:

$$∆Eab=\sqrt{{∆a}^{2}+{∆b}^{2}+{∆L}^{2}}$$

where ∆a, ∆b, and ∆L are the differences for L, a, and b values between sample and standard reference [23].

### 2.5. Nutritional Characterization

The nutritional characterization, including energy intake values proximate composition of the enriched pasta (P-AP), and the control pasta (P-CTR), was carried out by BonassisaLab S.p.a. (Foggia, Italy), an accredited food analysis laboratory. Fat content was determined using the acid hydrolysis method. Protein content was calculated as total nitrogen × 6.25. Total dietary fiber was measured using the AOAC (2000) method 985.29 [24], and carbohydrate content was calculated by subtracting the fiber fraction from the total carbohydrates.

#### 2.5.1. Starch Hydrolysis and Predicted Glycemic Index (pGI)

The predicted glycemic index (pGI) of P-CTR and P-AP pasta samples was determined using an in vitro model slightly modified from the method described by Canale et al. [25]. This method is based on starch hydrolysis and sugar release during digestion, as described by Goñi et al. [26]. Samples were pre-dried in a thermo-ventilated oven at 40 ± 1 °C for approximately 24 h until a constant weight was achieved and then ground into a fine flour using a coffee grinder (Moulinex, Lourdes, France). Briefly, 100 mg of dry pasta was digested sequentially. First, samples were digested with pepsin (Sigma-Aldrich P7125, 0.1 g/mL) in an HCl–KCl buffer (pH 1.5) at 40 °C for 1 h. After pepsin digestion, samples were incubated with α-amylase (Sigma-Aldrich A3176; 48 U mg/g of pasta) in Tris-Maleate buffer (pH 6.9) at 37 °C using an orbital shaker. During incubation, aliquots of 1 mL were removed at 0, 30, 60, 90, 120, 150, and 180 min. Each aliquot was heated at 100 °C for 5 min to inactivate the enzyme, cooled, and centrifuged at 10,000× *g* at 4 °C. For each supernatant, 500 µL was incubated with amyloglucosidase (330 U/mL) in 1.5 mL of sodium acetate buffer (pH 4.75) at 60 °C for 45 min. The glucose released during digestion was quantified using a Varioskan Flash Spectral Scanning Multimode Reader (Thermo Fisher Scientific, Waltham, MA, USA) at 510 nm, employing a commercial enzymatic kit (K-GLUC, Megazyme, Wicklow, Ireland) based on the glucose oxidase/peroxidase (GOPOD) enzyme system. The glucose release over time (0–180 min) was plotted to calculate the area under the curve (AUC). The hydrolysis index (HI) was determined as follows:

$$HI=\frac{AUC\;sample}{AUC\;reference}\times 100$$

White bread was used as the reference sample. The predicted glycemic index (pGI) was calculated using the formula:

pGI=39.71+0.549×HI

#### 2.5.2. Protein Solubility and Electrophoresis

The solubility of proteins in control and enriched pasta samples was determined in triplicate, as described by Bonomi et al. [27]. Briefly, finely ground samples (0.5 g) were dissolved in either 10 mL of 50 mM sodium phosphate buffer (pH 7.0) containing 0.1 M NaCl (buffer 1) or 10 mL of 50 mM sodium phosphate buffer (pH 7.0) containing 0.1 M NaCl, 8 M urea, and 10 mM dithiothreitol (DTT) (buffer 2). After stirring at room temperature for 60 min, samples were centrifuged at 10,000× *g* for 20 min at 15 °C to remove insoluble materials. The protein content in the supernatant was assessed using a colorimetric method [28], and solubility was inferred by comparing the extraction yield—calculated based on the protein concentration in the supernatant—between the two buffers used.

Ten µg of proteins extracted using buffer 2 was diluted with 15 µL of loading buffer (0.125 M Tris-HCl, pH 6.8, 20% glycerol, 2% SDS, 0.02% bromophenol blue, 5% 2-mercaptoethanol) and heated in boiling water for 10 min. SDS-PAGE analysis was performed on a 12% polyacrylamide gel using a Mini-PROTEAN apparatus (Bio-Rad, Hercules, CA, USA). Following staining with Coomassie Blue and destaining steps, proteins were visualized using a ChemiDocTM XRS+ (Bio-Rad), and images were analyzed with Image Lab^TM^ 5.1 Software (Bio-Rad).

### 2.6. Free and Bound Polyphenols Characterization

The free phenolic compounds were extracted from both cooked and uncooked P-CTR and P-AP pasta samples following the method reported by Bavaro et al. [10]. Briefly, polyphenols were extracted using ultrasounds with aqueous methanol (80% *v*/*v*) at matrix/solvent ratios of 1:5. The mixtures were sonicated (37 kHz, 50% potency, 30 °C, 10 min) with the Fisherbrand FB11203 ultrasonic bath, shaken (150 rpm, 20 min in the dark), centrifuged (4500× *g*, 10 min), and filtered at 0.45 µm. Exhaustive extraction was ensured by repeating the solvent addition, shaking, and combining the supernatants, which were stored at −20 °C. The residual pellets from both cooked and uncooked pasta samples were subjected to alkaline hydrolysis to extract bound polyphenols. This procedure involved treatment with 2 M NaOH for two hours, followed by acidification to pH 2 using 6 M HCl, and subsequently three successive extractions with ethyl acetate. The extracts were evaporated to dryness with a Rotavapor (BUCHI Italia s.r.l., Cornaredo, Italy), and then reconstituted in methanol (15% *v*/*v*) and filtered (0.45 µm).

Polyphenol profiles were analyzed by HPLC-DAD using the Agilent 1260 Infinity Series Chromatograph system, supplied with Agilent Open Lab CDS Chem Station Software version C.01.04 (Palo Alto, CA, USA). The system was equipped with 1260 HIP Degasser, G1312B binary pump, G1316A thermostat, and G4212B DAD detector. Separation was performed on a 5 μm Phenomenex Luna C18 (4.6 × 250 mm) column (Phenomenex, Torrance, CA, USA). The mobile phase consisted of MeOH (solvent A) and acetic acid/water (5:95 *v*/*v*) (solvent B) with the following gradient: 0–25 min, 15–40% A; 25–30 min, 40% A (isocratic); 30–45 min, 40–63% A; 45–47 min, 63% A (isocratic); 47–52 min, 63–100% A; 52–56 min, and 100% A (isocratic). The flow rate was constant at 1 mL/min. Main phenolic compounds were identified by comparing spectra and retention times with those of available standards. Results were expressed as mg of compound per 100 g of dry weight sample (DW).

### 2.7. Antioxidant Activity

Methanolic extracts of free and bound polyphenols from pasta samples were assayed to determine antioxidant activity using 2,2-diphenyl-1-pycrilhydracyl (DPPH), 2,2′-azino-bis(3-ethylbenzothiazoline-6-sulphonic acid) (ABTS), and ferric ion reducing antioxidant power (FRAP) assays. The radical scavenging activities were calculated using microplate methods with a Varioskan Flash Spectral Scanning Multimode Reader (Thermo Fisher Scientific).

The DPPH assay was performed as described by Brand-Williams et al. [29] by preparing a 1 mM solution of DPPH in methanol. For the calibration curve, Trolox was used at concentrations ranging from 0.005 mM to 1 mM. To determine antioxidant capacity, 20 µL of each methanolic extract or Trolox standard was added to 180 µL of the DPPH stock solution. After 30 min of incubation at room temperature in darkness, the absorbance was measured at 517 nm.

The ABTS free radical scavenging activity was performed as described by Re et al. [30]. A 7 mM ABTS stock solution was mixed with 2.45 mM potassium persulfate to obtain the ABTS cation radical solution, which was stored in darkness for 16 h prior to use. The ABTS solution was subsequently diluted to achieve an absorbance of approximately 0.700 at 734 nm. Then, 180 µL of the diluted solution was mixed with 20 µL of the extract or Trolox standard solution, and the absorbance was measured after 2 min at 734 nm.

The antioxidant capacity of extracts was also determined using the FRAP assay, as proposed by Benzie and Strain [31]. Known concentrations of ferrous sulfate solution (FeSO_4_), ranging from 0.1 to 1 mM, were used to prepare the calibration curve. The FRAP working reagent consisted of 300 mM acetate buffer (pH 3.6), 10 mM TPTZ (2,4,6-tripyridyl-s-triazine) in 40 mM HCl, and 20 mM FeCl_3_, in a ratio of 10:1:1 (*v*/*v*). The absorbance of the standards and each extract was measured at 593 nm after 4 min of incubation at 37 °C.

In all assays, the extracts were analyzed in triplicate. The results of the ABTS and DPPH tests were expressed as μmol of Trolox equivalents (TEs) per gram of dry weight (DW), while those for the FRAP assay were expressed as μmol of ferrous sulfate equivalents (FSEs) per gram of DW.

### 2.8. In Vitro Digestion and Polyphenol Bioaccessibility Evaluation

The bioaccessibility of polyphenols of pasta samples was assessed using a three-stage in vitro digestion model simulating the oral, gastric, and small intestinal phases, as described by Bavaro et al. [32], with minor modifications. Briefly, both control (P-CTR) and enriched (P-AP) pasta samples were cooked at their optimal cooking time (OCT) and subjected to simulated in vitro digestion. Cooked pasta (6 g) was mixed with oral phase solution (6 mL), saline solution (3 mL), and α-amylase (10.6 mg/g pasta), vortexed, and incubated at 37 °C with shaking (85 rpm) for 10 min. The gastric phase was initiated by adding a porcine pepsin solution (19 mg/mL in 0.1 M HCl) and adjusting the pH to 3.0 ± 0.1 using 1.0 M HCl. The solutions were then incubated at 37 °C, 85 rpm for 1 h. For the intestinal phase, a mixture containing pancreatin (30 mg/mL), lipase (15 mg/mL), and porcine bile salts (30 mg/mL) in 0.1 M NaHCO_3_ was added, and the solutions were incubated at 37 °C, 85 rpm for 2 h. After the small intestinal phase, the samples were centrifuged (10,000 rpm, 4 °C, 1 h). The supernatants were filtered (0.45 μm) and analyzed using HPLC-DAD to calculate the bioaccessibility of polyphenols. This was expressed as the percentage ratio of each phenolic compound released from cooked pasta in digested versus undigested samples.

The digested pasta pellets underwent bound polyphenol extraction to evaluate the colon-available index (CAI, %), as described by Lucas-González et al. [33].

### 2.9. Sensory Analysis

#### 2.9.1. Panel Test Design and Execution

Twelve expert evaluators with over 70 h of training in sensory analysis of a wide variety of foods, including pasta, performed the descriptive analysis (DA). Sensory tests were carried out at the IBE-CNR Sensory Lab (Bologna, Italy), in individual booths equipped with tablets running specific software for sensory data acquisition (FIZZ, Biosystemès, Couternon, France), according to the standard protocol UNI 8589:19901 [34]. Before executing the tests, participants were informed of the main research outcomes and gave consent for their data to be used. Participation in the research was voluntary, and the right to privacy and data protection was respected in accordance with current legislation (GDPR 2016/679) [35].

#### 2.9.2. Descriptive Analysis (DA)

Judges received 30 g of both samples, control (P-CTR) and enriched (P-AP), cooked at the optimal cooking time (OCT), in plastic plates labelled with three-digit random numbers. The samples were served on a tray at room temperature (20 ± 2 °C) and presented to assessors monadically, in a balanced order. Tests were carried out in duplicate and under the conditions described in the standard ISO 13299:2016 [36] for descriptive analysis using intensity scales (ISO 8586:2023) [37]. The sample evaluation order was randomized using a balanced Latin square design [38]. Twenty-five sensory attributes were selected from the literature [39] or added as new terms to create an appropriate lexicon list for the descriptive analysis. Five olfactory and six aromatic attributes were chosen as follows: wheat pasta, whole grain, vegetal, artichoke, legumes odor and flavor, and spicy tea flavor. Moreover, five gustatory and nine texture attributes were also evaluated as follows: sweet, acid, bitter, salty, umami; firmness, roughness, nerve, graininess, gummy, adhesiveness, fibrousness, flouriness, and astringency. The descriptors were rated on a 9-point scale from “no perception” to the “highest intensity perceivable”. The panelists were also asked to rate the products’ overall liking on a 9-point hedonic scale from “1: extremely disliked”; “5: neither liked nor disliked”; to “9: extremely liked”, as proposed for novel food by research [40]. Panelists used water to rinse their mouths between samples.

### 2.10. Statistical Analysis

The results were presented as mean values ± standard deviations. Data were subject to statistical analysis using Statistica 12.0 software (StatSoft, Inc., Tulsa, OK, USA). Data on physicochemical properties, polyphenol content, and antioxidant activity were compared using one-way ANOVA followed by Tukey’s test to determine significant differences (*p* < 0.05). Pearson’s correlation coefficient (r, *p* < 0.05) was calculated to assess correlations between antioxidant activities and polyphenol content. Sensory data were analyzed using IBM SPSS V. 27 and R programming language ver. 4.3.1 (R Core Team 2023. _R: A Language and Environment for Statistical Computing_. R Foundation for Statistical Computing, Vienna, Austria) and SensoMineR: Sensory Data Analysis for R package version 1.2. One-way ANOVA analysis was performed on DA sensory scores, and the Tukey post hoc test was carried out to test the differences between samples. The significance level was fixed at *p* < 0.05. Mean DA intensity values were used to generate a spider plot and represent the pasta sensory profiles.

## 3. Results and Discussion

The incorporation of artichoke powder (AP) into pasta formulation demonstrated notable impacts on its technological, nutritional, and functional characteristics.

### 3.1. Technological Properties

**Figure 1:**
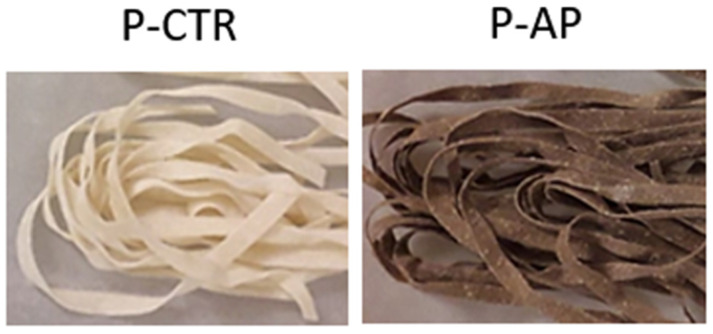
Evaluation of Fettuccine produced. P-CTR, pasta made with re-milled durum wheat semolina; P-AP pasta containing artichoke powder (10% *w*/*w* in substitution of semolina).

Table 1 summarizes the significant physicochemical and technological differences between control pasta (P-CTR) and enriched pasta (P-AP) produced. After drying, the Fettuccine obtained (Figure 1) showed water activity (a_w_) values of approximately 0.4. P-AP and P-CTR samples exhibited a similar optimal cooking time (OCT), indicating that the addition of AP did not significantly alter cooking performance (Table 1).

As previously observed [10], the replacement of 10% of semolina with AP, whose pH values (ca 5.1) are lower than those of re-milled durum wheat semolina (ca 6.2), determined a slight reduction in the pH of P-AP compared to the control samples (P-CTR). The titratable acidity of P-AP pasta, expressed as the volume (mL) of 0.1 M NaOH required to reach a pH value of 8.3, was also significantly higher than that of the control P-CTR, being 4.50 and 0.75, respectively. The raw materials used for pasta production presented different water-holding capacities; specifically, it was 1.1 and 6.6 g of water/g for re-milled durum wheat semolina and artichoke powder (AP), respectively. These results are consistent with findings reported in the literature [41]. The different absorption capacities of the ingredients used resulted in significantly (*p* < 0.05) higher water absorption at OCT in P-AP (+35% compared to P-CTR).

The results related to colorimetric differences between control pasta (P-CTR) and pasta enriched with artichoke powder (P-AP), analyzed using the CIE color system, showed that the incorporation of artichoke powder had a significant effect (*p* < 0.05) on pasta color. These changes are likely due to the natural pigments and polyphenols in artichokes, which influence color through oxidation and heat interactions. In particular, the enriched pasta showed a significant decrease in lightness (L*) compared to P-CTR. This darkening effect was expected, as artichoke powder contains natural pigments such as polyphenols, chlorophylls, and flavonoids, which contribute to a darker appearance. Additionally, the enriched pasta showed a higher a* coordinate, indicating a significant shift toward a more reddish coloration and a decrease in yellowness compared to the control sample [11]. Similar findings have been reported in previous studies on the incorporation of artichoke powder in baked products [10,15].

**Figure 2:**
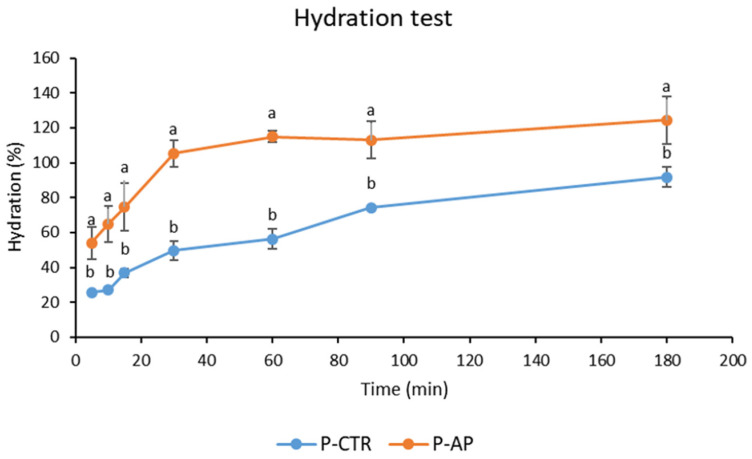
Kinetics of water absorption of control (P-CTR) and enriched (P-AP) pasta samples at 25 °C. ^a,b^ Values of the same time with different letters differ significantly (*p* < 0.05). Bars of standard deviations are also represented.

The kinetics of water absorption at 25 °C of pasta samples are reported in Figure 2. The substitution of semolina with 10% artichoke powder increased the water absorption of the pasta. Consequently, P-AP pasta showed faster and greater water absorption compared to P-CTR. Thus, the differing water absorption capacities of pasta samples were influenced by their distinct compositions, particularly the presence of hydrophilic macromolecules such as fibers, notably inulin, which is abundant in AP [10]. Finally, consistent with previous studies [42], cooking loss values for P-AP were higher (9.4 g/100 g pasta) than P-CTR (4.6 g/100 g), which could be attributed to the reduction in the gluten network in the P-AP dough.

### 3.2. Polyphenol Content and Antioxidant Activity

To maximize the benefits of a diet, it is essential to understand the presence of bioactive compounds, their distribution in food, and how food formulation, processing, and cooking can impact the availability of these beneficial components. Table 2 reports the characterization by HPLC-DAD of free phenolic compounds extracted from control (P-CTR) and enriched (P-AP) pasta samples before and after the cooking process. The results revealed a significant increase (*p* < 0.05) in polyphenol content in P-AP, where the most abundant phenolic compounds were chlorogenic acid, 3,5-dicaffeoylquinic acid, and 1,5-dicaffeoylquinic acid, accounting for 70% of the total identified polyphenols in P-AP. Flavonoids, including apigenin-7-O-glucoside, luteolin, and apigenin aglycone, were present at 17%. These results are consistent with our previous study on bread enriched with artichoke powder [10]. The main effect of processing was a reduction of about 42% in the free identified polyphenols due to cooking losses. After cooking, chlorogenic acid, 3,5-dicaffeoylquinic acid, and 1,5-dicaffeoylquinic acid remained the main identified compounds, followed by flavonoids. The reduction in polyphenol content after cooking has also been reported by other authors in studies on pasta enriched with different vegetable matrices [18,43,44].

Table 2 also reports polyphenol recovery after simulated digestion. Notably, there was an increase of approximately 47% in the total identified polyphenols compared to cooked P-AP pasta. This increase can be attributed to the release of bound polyphenols during digestion. To confirm this finding, the quantification and identification of bound polyphenols in P-AP pasta, compared to its control, were performed and are shown in Table 3.

The polyphenol content was found to be 19.41 mg/100 g DW, a value comparable to the increase observed after digestion (15 mg/100 g DW). The main identified compounds bound to the artichoke powder were caffeic acid, ferulic acid, apigenin, and coumaric acid. In particular, caffeic acid accounted for 66.5% of the total identified compounds, followed by nearly 4 mg/100 g DW of ferulic acid—a typical wheat flour compound also present in the control (P-CTR)—1.19 mg/100 g DW of apigenin-7-O-glucoside, and 1.1 mg/100 g DW of coumaric acid. In P-AP pasta, the flavonoid apigenin accounted for approximately 6.1%, and the obtained results are comparable to those reported for bread enriched with the same artichoke powder [10]. After digestion, only 3% of bound polyphenols were recovered in the fraction used to calculate the colon availability index (CAI). This parameter represents the polyphenols not released in the upper GI tract but available in the colon for further microbial metabolism [33]. The highest CAI was observed for apigenin, including both apigenin-7-O-glucoside and apigenin (44%). It is also interesting to note the presence of luteolin aglycone, albeit in small amounts, which is directly available for colonic metabolism. Several authors have reported that polyphenols reaching the colon undergo biochemical transformations such as hydrolysis, cleavage, reduction, and deglycosylation, leading to the production of low-molecular-weight derivatives. These compounds, through their interaction with gut microbiota, may enhance bioavailability and provide additional health benefits [5,45,46]. Recently, Cheng et al. [47] explained how bioactive compounds such as polyphenols, despite their low bioavailability, can exert significant health benefits. The authors suggest that polyphenols influence and regulate gut microbiota composition by promoting microbial metabolism, leading to the production of trimethylamine N-oxide (TMAO) and short-chain fatty acids (SCFAs). Additionally, they are metabolized into more bioavailable compounds with enhanced bioactivity [47].

Table 4 presents the antioxidant capacity of polyphenol extracts from uncooked, cooked, digested, and bound P-AP pasta compared to the control (P-CTR). The following three different methods were used: the ABTS and DPPH assays, which are most suitable for evaluating the radical-scavenging power of artichoke-enriched pasta, and the FRAP assay, which assesses the ability of artichoke polyphenols to counteract heavy metals involved in free radical production. These tests are primarily based on electron transfer to evaluate the capacity of polyphenols to scavenge free radicals through electron donation. In the DPPH assay, hydrogen atom transfer is also involved. For the ABTS and DPPH assays, results are expressed as µmol of Trolox equivalents/g DW, while for the FRAP assay, results are expressed as µmol of ferrous sulfate equivalent/g DW.

The results showed that enriched pasta (P-AP) had significantly higher (*p* < 0.05) antioxidant capacity values across all assays. Specifically, the free polyphenol extracts from uncooked P-AP exhibited the highest antioxidant capacity, both in terms of radical scavenging and the ability to counteract heavy metal reduction (Fe^3+^ to Fe^2+^), compared to the control. Specifically, the highest value was recorded for ABTS (22.26 µmol TE/g DW), followed by DPPH (4.67 µmol TE/g DW) and FRAP (4.28 µmol FSE/g DW). Although the FRAP method has been reported to have a low biological correlation with polyphenol antioxidant activity [48], combining multiple antioxidant assays helps provide a more comprehensive evaluation. Processing reduced the antioxidant capacity of free polyphenol extracts, but the trend remained similar to that observed in uncooked pasta (Table 4), with P-AP maintaining a higher antioxidant level than the control. Similar results were reported by la Gatta et al. [18] for pasta enriched with a low percentage (3%) of lyophilized artichoke waste. Polyphenols in food matrices such as pasta are also present in bound forms. In this study, the antioxidant power of bound polyphenol extracts from P-AP was assessed, considering both cooking and digestive conditions. As observed for free compounds, the highest antioxidant capacity for bound polyphenol extracts was recorded using the ABTS assay (6.99 µmol TE/g DW), followed by FRAP (1.91 µmol FSE/g DW) and DPPH (1.65 µmol TE/g DW). After cooking and digestion, the highest values in P-AP samples were recorded for FRAP (0.393 µmol FSE/g DW), followed by ABTS (0.328 µmol TE/g DW) and DPPH (0.086 µmol TE/g DW).

Moreover, the values obtained from the ABTS assay were higher than those recorded using the DPPH assay, a trend also observed in other studies evaluating the oxidative capacity of plant-based matrices [15,49]. As explained by Sadowska-Bartosz and Bartosz [50], it is recommended to use multiple assays, as each measures different pools of antioxidants in complex food matrices. Additionally, Pearson’s correlation coefficients were used to evaluate the relationship between polyphenol content and antioxidant capacity. A statistically significant (*p* < 0.05) positive correlation was found across all assays, with r values of 0.99, 0.95, and 0.90 for DPPH, ABTS, and FRAP, respectively. These results confirm the role of artichoke by-products as rich sources of antioxidants and demonstrate that the polyphenols recovered from each extract contribute to the antioxidant activity of pasta samples.

### 3.3. Nutritional Properties

#### 3.3.1. Proximate Composition

Pasta generally contains a high amount of starch and low levels of health-promoting compounds such as dietary fiber, minerals, vitamins, and polyphenols. Several studies have proposed improving the nutritional value of pasta by incorporating functional ingredients, often derived from agri-food by-products, to enhance physiological benefits and reduce disease risks [11,51,52]. The functional ingredients added to pasta mainly include dietary fiber, proteins, omega-3 fatty acids, and polyphenols, whose consumption has been associated with various health benefits, including antidiabetic, antioxidant, anti-inflammatory, and antibiotic effects. As reported in Table 5, few significant differences (*p* > 0.05) were observed in the analyzed nutritional parameters among pasta samples. In fact, both samples showed similar energy values (*p* > 0.05), with P-CTR at 1500 KJ (354 Kcal) and P-AP at 1487 KJ (351 Kcal). Notably, P-AP exhibited a significantly higher dietary fiber content (9.8%) compared to P-CTR (2.8%), contributing to its improved health profile.

Moreover, the fiber content in the enriched pasta allows the product to be classified as HIGH FIBER, as it exceeds the 6% dietary fiber threshold required by the nutrition claims listed in the Annex of Regulation (EC) No. 1924/2006 [53], as last amended by Regulation (EU) No. 1047/2012 [54]. It is also noteworthy that both P-AP and its control (P-CTR) are rich in mono- and polyunsaturated fatty acids, likely deriving from semolina, confirming the importance of consuming this cereal-based food. Furthermore, the presence of dietary fiber and phenolic compounds in the enriched pasta contributes to reducing the glycemic index compared to the control, as shown in Table 5. Specifically, P-AP had a lower predicted glycemic index (pGI) of 56.67 compared to 58.41 for P-CTR, representing a significant benefit in modulating blood glucose levels. The reported pGI is also lower than that reported by la Gatta et al. [18] for artichoke-enriched pasta, although the authors used a lower enrichment percentage (3%) compared to the present study.

The findings presented here align with previous studies highlighting the role of dietary fiber, particularly artichoke by-products, in moderating glycemic response, as seen in bread enriched with artichoke powder or pasta with chicory inulin [10,32].

#### 3.3.2. Protein Solubility and Electrophoretic Migration

**Figure 3:**
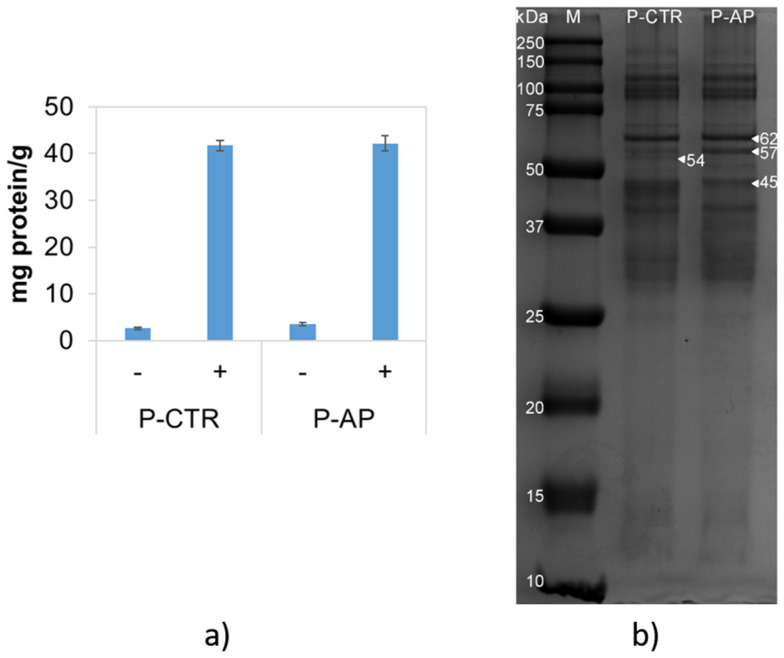
Solubility of proteins in control (P-CTR) and enriched (P-AP) pasta samples with different extraction buffers (**a**). − and + indicate either the absence or the presence of urea and DTT. SDS-PAGE analysis of proteins from control (P-CTR) and enriched (P-AP) pasta samples (**b**).

The rheological and textural properties of pasta are affected by the constituents of wheat flour. Particularly, proteins may form insoluble aggregates via hydrophobic interactions and disulfide bonds, influencing elasticity and adhesiveness [55]. This study of protein solubility provides insights into the amount of proteins able to aggregate. Protein solubility was assessed in both P-CTR and P-AP samples using two extraction buffers (Figure 3a). No differences were observed between the two samples. The addition of denaturing and reducing agents, such as urea and DTT, resulted in a clear increase in protein solubility—approximately 10-fold—compared to the standard buffer. This suggests the crucial role of hydrophobic interactions and of disulfide bonds in stabilizing insoluble protein aggregates [55]. SDS-PAGE analysis of proteins extracted in the presence of urea and DTT revealed slight differences in protein composition between the two samples, particularly in the range of 40–60 kDa (Figure 3b). P-AP exhibited a higher abundance of the 62 and 57 kDa bands, while it lacked the 54 kDa band and displayed a lower level of the 45 kDa band. These bands could correspond to glutenin(s), which are closely associated with adhesiveness [55]. Indeed, the observed differences suggest a possible modification in the protein structure of P-AP pasta that may result from interaction between wheat proteins and bioactive compounds from the artichoke powder.

The analysis of bound polyphenols in enriched pasta was conducted to evaluate the influence of gluten on polyphenol release from the dough, given the strong interactions between phenolics and proteins [56]. Dietary polyphenols, such as chlorogenic acid present in artichoke powder, can bind to gluten, reducing its digestibility and immunogenicity due to the presence of quinic acid in its structure. This interaction may suggest a potential role of polyphenols in modulating gluten digestion and adsorption, which could be relevant for individuals with celiac disease [57].

### 3.4. Descriptive Analysis Profile Results

**Figure 4:**
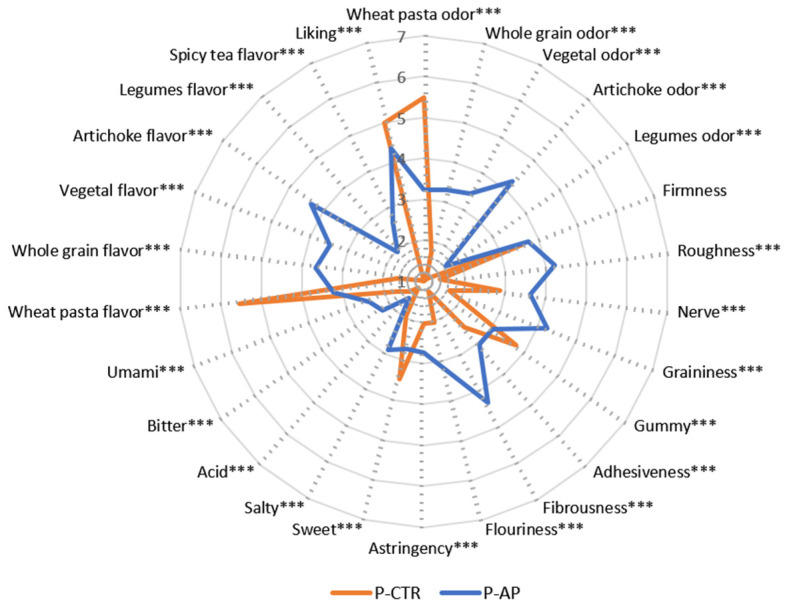
DA profiles of the control (P-CTR) and enriched (P-AP) pasta. Mean intensity scores for the sensory attributes of two pasta samples, according to one-way ANOVA followed by Tukey post hoc test (*** *p* < 0.001).

Trained panel sensory data highlighted clear sensory differences between the samples (Figure 4), with PA enrichment significantly impacting the pasta’s aromatic profile. The control sample (P-CTR) exhibited a higher intensity only for wheat pasta odor and flavor, whereas the enriched sample (P-AP) showed a higher intensity for most of the perceived odors and flavors, including whole grain, vegetal, artichoke, legume, and spicy tea flavors. This is in accordance with la Gatta et al. [18], who demonstrated the influence of a powder derived from artichoke outer bracts in fresh pasta formulation on its volatile profile. The P-CTR sample was sweeter while exhibiting lower intensity in umami, salty, acidic, bitter, and astringent sensations. The slightly higher astringency and bitterness of P-AP can be attributed to the presence of cynaropicrin in the artichoke [15,58].

Further, in agreement with the technological properties, fiber enrichment in the pasta sample containing artichoke powder (P-AP) proved to influence the structural integrity of the pasta during cooking, enhancing texture attributes such as roughness, nerve, graininess, adhesiveness, fibrousness, and flouriness. The control sample (P-CTR) recorded the highest intensity for gumminess. These findings align with the differences observed between the two pasta samples in protein composition.

Judges expressed their highest appreciation for the control sample (5.0), likely due to familiarity, as consumers tend to appreciate products they are more familiar with. The familiarity and cultural significance of traditional foods, such as regular pasta, make it challenging for consumers to accept new or enriched variants [59]. Despite that, the enriched pasta received an encouraging evaluation (4.3) and may increase in acceptance by providing information about naturalness and health benefits. Recent studies demonstrate that consumers are not willing to swap potential health benefits for hedonic attributes [60,61], and those who are more taste-oriented are skeptical about new product formulas [62]. However, their opinion now is changing, and the adequate information on the health benefits has the potential to increase consumers’ inclination toward and acceptance of novel functional foods [2].

## 4. Conclusions

This study highlights the feasibility of enriching pasta with artichoke powder to improve its nutritional profile and functional properties while addressing food waste concerns. The inclusion of 10% AP led to significant improvements in polyphenol content, antioxidant capacity, and dietary fiber levels. The in vitro digestion study confirmed the bioaccessibility of these bioactive compounds, suggesting potential health benefits, particularly in gut health and metabolic regulation.

From a technological perspective, the increased water absorption and cooking loss indicate that fiber enrichment alters pasta structure, which may require optimization to maintain optimal texture. Sensory analysis revealed that P-AP had distinct vegetal and bitter notes, which may influence consumer acceptance. However, the growing consumer demand for functional foods could enhance the market potential of enriched pasta. Descriptive analysis has allowed us to gain an in-depth understanding of the sensory profile of both enriched and non-enriched pasta, helping to predict consumer appreciation.

Overall, the results demonstrate that artichoke bracts can be effectively upcycled into a value-added food ingredient, supporting sustainable food production. Future research should explore formulation adjustments and consumer acceptance strategies to maximize the appeal of enriched pasta while retaining its health benefits.

## Figures and Tables

**Table 1:** Physicochemical and technological characteristics of control (P-CTR) and enriched (P-AP) pasta samples.

	P-CTR	P-AP
pH	6.11 ± 0.07 ^a^	5.89 ± 0.06 ^b^
TTA (mL) *	0.75 ± 0.07 ^b^	4.50 ± 0.07 ^a^
OCT (min)	6.5 ± 0.01 ^a^	6.5 ± 0.01 ^a^
Water absorption (%)	128.26 ± 10.95 ^b^	173.04 ± 4.53 ^a^
Cooking loss (g/100 g pasta)	4.6 ± 0.28 ^b^	9.4 ± 0.85 ^a^
Color analysis		
∆L	53.67 ± 0.26 ^a^	33.88 ± 0.54 ^b^
∆a	1.05 ± 0.48 ^b^	3.30 ± 0.16 ^a^
∆b	20.78 ± 1.02 ^a^	16.26 ± 0.45 ^b^
∆Eab	57.57 ± 0.38 ^a^	37.73 ± 0.52 ^b^

Data represent means of three independent experiments ± standard deviations. ^a,b^ Values in the same row with different letters differ significantly (*p* < 0.05). * Total titratable acidity (TTA) values of pasta samples were expressed in mL of 0.1 N NaOH required to achieve a pH of 8.3.

**Table 2:** Free polyphenol characterization by HPLC-DAD of uncooked, cooked, and digested control (P-CTR) and enriched (P-AP) pasta samples.

		Pasta					
Rt	Polyphenols	P-CTR			P-AP		
(min)	(mg/100 g DW)	Uncooked	Cooked	Digested	Uncooked	Cooked	Digested
4.37	1-O-caffeoylquinic acid	nd	nd	nd	1.491 ± 0.075 ^b^	1.193 ± 0.060 ^c^	1.968 ± 0.098 ^a^
4.76	3-O-caffeoylquinic acid	nd	nd	nd	0.859 ± 0.043 ^b^	0.716 ± 0.036 ^c^	1.076 ± 0.054 ^a^
7.12	Chlorogenic acid	nd	nd	nd	14.998 ± 0.750 ^a^	9.310 ± 0.465 ^b^	14.025 ± 0.701 ^a^
14.23	Coumaric acid	0.038 ± 0.002 ^d^	0.023 ± 0.001 ^d^	0.107 ± 0.005 ^c^	0.179 ± 0.009 ^b^	0.096 ± 0.005 ^c^	0.379 ± 0.019 ^a^
14.81	Apigenin derivative	0.139 ± 0.007 ^d^	0.054 ± 0.003 ^e^	0.193 ± 0.010 ^d^	0.526 ± 0.026 ^b^	0.295 ± 0.015 ^c^	1.034 ± 0.052 ^a^
16.63	Ferulic acid	0.325 ± 0.016 ^a^	0.131 ± 0.007 ^c^	0.353 ± 0.018 ^a^	0.235 ± 0.012 ^b^	0.271 ± 0.014 ^b^	0.265 ± 0.013 ^b^
16.90	1,4-dicaffeoylquinic acid	nd	nd	nd	1.441 ± 0.072 ^b^	0.664 ± 0.033 ^c^	2.238 ± 0.112 ^a^
17.74	4,5-dicaffeoylquinic acid	nd	nd	nd	0.852 ± 0.043 ^b^	0.648 ± 0.032 ^c^	1.504 ± 0.075 ^a^
18.37	3,5-dicaffeoylquinic acid	nd	nd	nd	7.433 ± 0.372 ^a^	3.875 ± 0.194 ^c^	5.182 ± 0.259 ^b^
19.47	1,5-dicaffeoylquinic acid	nd	nd	nd	15.185 ± 0.759 ^a^	8.428 ± 0.421 ^c^	12.466 ± 0.623 ^b^
22.96	3,4-dicaffeoylquinic acid	nd	nd	nd	1.552 ± 0.078 ^b^	1.196 ± 0.060 ^c^	2.019 ± 0.101 ^a^
24.21	Apigenin-7-O-glucoside	nd	nd	nd	5.008 ± 0.250 ^a^	2.786 ± 0.139 ^c^	3.738 ± 0.187 ^b^
34.88	Luteolin	nd	nd	nd	1.003 ± 0.050 ^a^	0.648 ± 0.032 ^b^	0.341 ± 0.017 ^c^
40.83	Apigenin	nd	nd	nd	2.801 ± 0.140 ^a^	2.148 ± 0.107 ^b^	1.212 ± 0.061 ^c^
	Total	0.502 ± 0.025 ^d^	0.208 ± 0.010 ^d^	0.653 ± 0.033 ^d^	53.563 ± 2.678 ^a^	32.275 ± 1.614 ^c^	47.447 ± 2.372 ^b^

Data represent means of three independent experiments ± standard deviations. ^a–e^ Values in the same row with different letters differ significantly (*p* < 0.05). *Rt*: retention time. nd: not detected.

**Table 3:** Bound polyphenol characterization by HPLC-DAD of CTR and AP pasta samples.

		Pasta			
*Rt*	Polyphenols	P-CTR		P-AP	
(min)	(mg/100 g DW)	Uncooked	Digested	Uncooked	Digested
9.33	Caffeic acid	nd	0.028 ± 0.001 ^b^	11.968 ± 0.598 ^a^	nd
14.27	Coumaric acid	0.127 ± 0.006 ^b^	0.006 ± 0.001 ^c^	1.099 ± 0.055 ^a^	nd
16.55	Ferulic acid	4.960 ± 0.248 ^a^	0.091 ± 0.005 ^c^	4.225 ± 0.211 ^b^	0.019 ± 0.001 ^c^
24.35	Apigenin 7-O-glucoside	nd	nd	1.188 ± 0.059 ^a^	0.084 ± 0.004 ^b^
30.10	Caffeic acid derivative 1	0.187 ± 0.009 ^b^	nd	0.496 ± 0.025 ^a^	nd
31.67	Caffeic acid derivative 2	0.210 ± 0.010 ^b^	nd	0.436 ± 0.022 ^a^	nd
34.90	Luteolin	nd	nd	nd	0.021 ± 0.001 ^a^
40.90	Apigenin	nd	nd	nd	0.445 ± 0.022 ^a^
	Total	5.483 ± 0.274 ^b^	0.125 ± 0.006 ^c^	19.411 ± 0.971 ^a^	0.570 ± 0.028 ^c^

Data represent means of three independent experiments ± standard deviations. ^a–c^ Values in the same row with different letters differ significantly (*p* < 0.05). *Rt*: retention time. nd: not detected.

**Table 4:** Antioxidant activity of extracts obtained from uncooked, cooked, bound polyphenols, and digested control (CTR) and enriched (AP) pasta samples.

	DPPH		ABTS		FRAP	
	(μmol TE/g DW)		(μmol TE/g DW)		(μmol FSE/g DW)	
Pasta Extract	P-CTR	P-AP	P-CTR	P-AP	P-CTR	P-AP
Free polyphenols						
Uncooked	nd	4.627 ± 0.766 ^a^	6.618 ± 0.093 ^b^	22.263 ± 1.191 ^a^	0.766 ± 0.023 ^b^	4.278 ± 0.199 ^a^
Cooked	nd	3.616 ± 0.367 ^a^	4.021 ± 0.031 ^b^	13.505 ± 2.491 ^a^	0.428 ± 0.008 ^b^	3.154 ± 0.064 ^a^
Bound polyphenols						
Uncooked	0.264 ± 0.016 ^b^	1.647 ± 0.296 ^a^	1.923 ± 0.009 ^b^	6.992 ± 0.086 ^a^	0.416 ± 0.003 ^b^	1.907 ± 0.081 ^a^
Cooked	0.041 ± 0.008 ^b^	0.086 ± 0.015 ^a^	0.245 ± 0.006 ^a^	0.328 ± 0.059 ^a^	0.205 ± 0.006 ^b^	0.393 ± 0.009 ^a^

Data represent means of three independent experiments ± standard deviations. ^a,b^ Values in the same row within each column with different letters differ significantly (*p* < 0.05). TE: Trolox equivalent. FSE: ferrous sulfate equivalent.

**Table 5:** Average nutritional values of CTR and AP pasta.

	Pasta	
Nutrient Content	P-CTR	P-AP
Energy (KJ/Kcal)/100 g	1500 ± 80/354 ± 19 ^a^	1487 ± 70/351 ± 17 ^a^
Fat (%)	1.09 ± 0.05 ^a^	1.4 ± 0.3 ^a^
of which: saturates	0.12 ± 0.02 ^b^	0.28 ± 0.07 ^a^
Carbohydrate (%)	71.5 ± 4.4 ^a^	68.1 ± 3.8 ^a^
of which: sugars	2.57 ± 0.71 ^a^	2.41 ± 0.68 ^a^
Fiber (%)	2.8 ± 0.5 ^b^	9.8 ± 0.9 ^a^
Protein (%)	13.03 ± 1.67 ^a^	11.64 ± 1.39 ^a^
Saturated fatty acids (%)	11.13 ± 1.57 ^b^	19.43 ± 2.48 ^a^
Monounsaturated fatty acids (%)	24.58 ± 3.32 ^a^	18.22 ± 2.44 ^a^
Polyunsaturated fatty acids (%)	64.29 ± 8.68 ^a^	62.35 ± 7.79 ^a^
Hydrolysis Index (HI)	71.78 ± 0.13 ^a^	70.82 ± 0.28 ^b^
Predicted glycemic index (pGI)	58.41 ± 0.24 ^a^	56.67 ± 0.52 ^b^

Data represent means of three independent experiments ± standard deviations. ^a,b^ Values in the same row with different letters differ significantly (*p* < 0.05).

## Data Availability

Raw data concerning the profiling assays are available on request to the corresponding author.

## Abbreviations

The following abbreviations are used in this manuscript:

AP	artichoke powder
AUC	area under the curve
a_w_	water activity
DW	dry weight
FSE	ferrous sulfate equivalent
HI	hydrolysis index
OCT	optimal cooking time
P-AP	enriched pasta with artichoke powder
P-CTR	control pasta
pGI	predicted glycemic index
TE	Trolox equivalent
TTA	total titratable acidity
